# Prof T K Shanmugasundaram (1929–2008)

**Published:** 2008

**Authors:** S Rajasekaran

**Affiliations:** *Ganga Hospital, Coimbatore, India E-mail: Rajasekaran.orth@gmail.com*

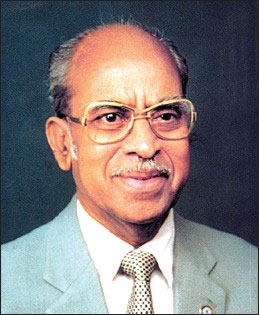



The 11^th^ day of August, 2008 saw curtains close on the distinguished life of one of the giants in the field of orthopedics, traumatology, and spine surgery. Prof. T. K. Shanmugasundaram passed away after a brief illness. His death has left a void in the hearts of numerous students for whom he was a constant source of inspiration.

Prof. TKS (as he was more popularly known amidst his orthopedic peers worldwide) was born on 8th August 1929 to an illustrious family as one of seven children. He always held his parents in such high esteem that in his autobiography, ‘All my yesterdays,’ he included a considerable chapter on his father prefacing his autobiography, stating that, ‘I feel that a life sketch of my father is most appropriate… for his contributions far outweigh those of his children..…’

He obtained his MBBS degree from Stanley Medical College in 1953 and subsequently got an MS in 1957, FRCS (Edinburgh and England) both in 1962, and the MCh (Orth) in Liverpool in 1963. After his training in the UK, he played a considerable and significant role in the development of the orthopedic specialty in South India, first heading the department in Stanley Medical College, and then later, as Professor and Head of the Department in Madras Medical College from 1976 to 1981. He was also the Director of the Government Institute of Rehabilitation and Artificial Limb Centre (1979–1983), the principal investigator and project director of the famous Madras study of tuberculosis of the spine (a collaborative project of ICMR, India and MRC, Great Britain) (1976–1987), and the principal investigator and Project Director of PL.480 Paraplegia project and ICMR Paraplegia Project (1979–1987). He was the postgraduate examiner for over 20 universities, for the National Board of Examinations in India and abroad. His authority in the field made him a popular guest speaker and orator who was invited for many prestigious orations, both nationally and internationally. He also had the distinction of being the President of the Tamil Nadu Orthopedic Association for three years (1973–1976); President of the Indian Orthopedic Association (1982); President-World Orthopedic Concern (1996–99), Chairman-Coordinator, International Bone and Joint Tuberculosis Club, and Chairman and Vice-President, Indian Red Cross Society, Tamil Nadu. In 1998, he was honoured with a ‘Honorary’ Doctor of Science degree by The Tamil Nadu Dr. M. G. R. Medical University.

Apart from being a renowned surgeon, Prof. TKS was most respected for being an excellent teacher and a beacon of ethical practice. In his long tenure as an orthopedic teacher for over 30 years, he taught over 4000 undergraduate medical students and 150 postgraduate students. Apart from learning orthopedics, his students could learn much more in the way of ‘invisible curriculae’ from him. He was an outstanding role model for punctuality, discipline, and patient care. During his ward rounds, he would scarcely hesitate to publicly admonish anyone, regardless of their seniority, if any carelessness or callousness was found in patient care. His compassion towards poor patients was always exemplified by the long time he spent on rounds in the general wards whereas he would quickly pass the special wards which housed the rich and the influential, even some ministers in power.

Prof. TKS was an embodiment of ethical values to the extent that he was a ‘second conscience’ to all of his students. He was a standing example of how success could be achieved without mixing academic and private practice. Prof. TKS was always against practices of overprescription, overinvestigation, professional kickbacks, and dichotomy of fees which earned him great esteem in the minds and hearts of generations of students. He was true to the sayings of Mahatma Gandhi, ‘Parents are your best teachers; and teachers are your second parents,’ and himself was a source of great learning and discipline for all his students.

His love for ‘academic truth’ was also legendary and he was never one to shy away from presenting his complications. One could easily see him shrink with horror and despair when he noticed senior members presenting distorted facts and statistics and would not hesitate to publicly admonish them. Among his peers, he stood tall as an embodiment of ethics and honesty.

Prof. TKS was a torch bearer of academics and research. His clinical research on bone and joint tuberculosis, spinal injury care, bone tumors, and post-injection fibrosis of skeletal muscles were presented in over 80 national and international conferences and published in nearly 80 papers in national and international journals. However, he always placed patient care first and stood as an example to his own dictum that in a clinician's life, the three important things are patient care, teaching, and research, strictly in that order. He would excuse a postgraduate for a little delay in academic commitments, but never in matters relating to patient care.

Prof. TKS was also an intense family man. His love for his wife, Uma, was well-known and he could never seriously come out of his shock after her demise a few years ago. The poem he wrote on the occasion of her death, ‘The Bell rings no more,’ showcased his deep love for her and portrayed the tenderness in his heart which he rarely revealed in public. He is survived by his son, Vignesh, and three daughters, Vijayalakshmi, Shalini, and Nandini.

His death has left an irreplaceable void in this part of the orthopedic world and he will always be missed by his students, colleagues, and friends throughout the world. His life was one of strict discipline, hard work, honesty, plain living, and high thinking, and was one that could be emulated by the younger generation. His students are privileged to have been trained by such an extraordinary academician, teacher, and a noble hearted individual as Prof. TKS.

*'Lives of great men all remind us*
*We can make our lives sublime*
*And departing, leave behind us*
*Footprints on the sands of time'*
H. W. Longfellow

